# Narratives Reflecting the Lived Experiences of People with Brain Disorders: Common Psychosocial Difficulties and Determinants

**DOI:** 10.1371/journal.pone.0096890

**Published:** 2014-05-07

**Authors:** Sally Hartley, Maggie McArthur, Michaela Coenen, Maria Cabello, Venusia Covelli, Joanna Roszczynska-Michta, Tuuli Pitkänen, Jerome Bickenbach, Alarcos Cieza

**Affiliations:** 1 Faculty of Medicine and Health Sciences, University of East Anglia, Norwich, United Kingdom; 2 Faculty of Health Sciences, University of Sydney, Sydney, New South Wales, Australia; 3 The London School of Hygiene and Tropical Medicine, London, United Kingdom; 4 Department of Medical Informatics, Biometry and Epidemiology – IBE, Chair for Public Health and Health Services Research, Research Unit for Biopsychosocial Health, Ludwig-Maximilians-University (LMU), Munich, Germany; 5 Instituto de Salud Carlos lll CIBERSAM, Faculty of Medicine, Universidad Autonoma de Madrid, Instituto de investigacion de la Princesa (IIS-IP), Madrid, Spain; 6 Neurology, Public Health and Disability Unit, Scientific Directorate, Neurological Institute Carlo Besta IRCCS Foundation, Milan, Italy; 7 Institute of Neurology and Psychiatry, Warsaw, Poland; 8 A-Clinic Foundation (A-klinikkasäätiö), Helsinki, Finland; 9 Swiss Paraplegic Research (SPR), Nottwil, Switzerland; 10 Faculty of Social and Human Sciences, University of Southampton, Southampton, United Kingdom; The University of Edinburgh, United Kingdom

## Abstract

**Background:**

People with brain disorders - defined as both, mental disorders and neurological disorders experience a wide range of psychosocial difficulties (PSDs) (e.g., concentrating, maintaining energy levels, and maintaining relationships). Research evidence is required to show that these PSDs are common across brain disorders.

**Objectives:**

To explore and gain deeper understanding of the experiences of people with seven brain disorders (alcohol dependency, depression, epilepsy, multiple sclerosis, Parkinson’s disease, schizophrenia, stroke). It examines the common PSDs and their influencing factors.

**Methods:**

Seventy seven qualitative studies identified in a systematic literature review and qualitative data derived from six focus groups are used to generate first-person narratives representing seven brain disorders. A theory-driven thematic analysis of these narratives identifies the PSDs and their influencing factors for comparison between the seven disorders.

**Results:**

First-person narratives illustrate realities for people with brain disorders facilitating a deeper understanding of their every-day life experiences. Thematic analysis serves to highlight the commonalities, both of PSDs, such as loneliness, anger, uncertainty about the future and problems with work activities, and their determinants, such as work opportunities, trusting relationships and access to self-help groups.

**Conclusions:**

The strength of the methodology and the narratives is that they provide the opportunity for the reader to empathise with people with brain disorders and facilitate deeper levels of understanding of the complexity of the relationship of PSDs, determinants and facilitators. The latter reflect positive aspects of the lives of people with brain disorders. The result that many PSDs and their influencing factors are common to people with different brain disorders opens up the door to the possibility of using cross-cutting interventions involving different sectors. This strengthens the message that ‘a great deal can be done’ to improve the lived experience of persons with brain disorders when medical interventions are exhausted.

## Introduction

People living with a brain disorder experience a range of psychosocial difficulties (PSDs) [1]–[5]. Health professionals treating people with these brain disorders within their specialties recognize that coping with these PSDs are the major challenge in the life of their patients.

Brain disorders include both “mental disorders”, such as depression, schizophrenia, and substance dependence and neurological disorders, such as Parkinson’s disease, stroke, dementia. Both groups together are also frequently referred to as neuropsychiatric disorders. Only more recently they are more comprehensively denoted as “disorders of the brain” or brain disorders [6]. PSDs refer to impairments of body functions under central nervous system control, activity limitations and participation restrictions, such as concentrating, sleeping, maintaining energy levels, anxiety, making and maintaining relationships and keeping a job. Based on the conceptualization of the International Classification of Functioning, Disability and Health (ICF) [7], PSDs are the result of an interaction of the health condition, environmental and personal factors.

Looking across the disorder-specific literature it can easily be seen that a large number of those PSDs are common. However, “…*lay persons and professionals are typically unaware of the commonalities and the shared mechanisms of ‘brain disorders” (pp 656)* [6]. Since research and service provision are framed around medical diagnoses, single brain disorders or simple combinations of one or two of them [8], researchers and health professionals tend to specialize accordingly. Even rehabilitation health professionals, who are initially trained to think across and beyond health conditions, eventually specialize in specific conditions in order to gain credibility in the research arena or to fit within a medically framed service structure [9].

Looking at commonalities in PSDs across brain disorders has the potential for specialists to learn from one another for the benefit of their patients. For example, we need to know whether the intervention shown to be effective for addressing fatigue in people with multiple sclerosis is also effective for people with other brain disorders such depression or stroke. The recognition that PSDs are common across brain disorders also challenges the premise that the medical diagnosis has to be the driver of care for these people. Literature shows that, even though getting a diagnosis gives some sense of relief [10], what people really care about is to be able to continue with their lives [11]. It has been shown that an approach of care that addresses the difficulties faced by people with different brain disorder diagnoses can be effective [12].

The perspective of commonalities across brain disorders is also supported by the conceptualization of health and disability found in the World Health Organization’s (WHO) ICF. Based on this conceptualization, the difficulties people with health conditions face in their lives do not only relate to their health conditions but also to personal factors and the context in which they live. It is for this reason that two people with the same condition may experience different kinds of PSDs while two people with different conditions may experience the same PSDs.

The claim, however, that there is a horizontal perspective of PSDs across brain disorders currently is theoretical and inferred from the condition-specific clinical research literature and the experience of health professionals. Research evidence is required to support this claim holds. We propose that the first evidence needed should be based on the actual experience of people living with these brain disorders and the most appropriate methodology to understand their perspective is by using qualitative research methodology [13].

We want to extend the claim to include the factors which influence PSDs. The arguments for this are similar to those already mentioned, namely, the condition-specific literature independently reports similar determinants for PSDs across brain disorders, for example, the influence of social attitudes for getting or maintaining a job is highlighted in the literature related to people with schizophrenia, stroke and epilepsy [14]–[16].

This investigation seeks to explore and gain deeper understanding of the experiences of people with different brain disorders using available qualitative data. It examines the common PSDs and their influencing factors. The research questions addressed are: (1) What is the nature of the lived experience of people with brain disorders? (2) What are the PSDs associated with these experiences? (3) What are the common PSDs? (4) What are the facilitators of and barriers to these PSDs?

## Methods

The brain disorders addressed in this study are alcohol dependency, depression, epilepsy, multiple sclerosis, Parkinson’s disease, schizophrenia, and stroke. These conditions were selected because, together with migraine and dementia, they were focused on in the EU-funded project PARADISE from which the data was drawn. The overall aim of PARADISE was to seek for evidence to support the claim that there is a considerable degree of commonality in PSDs across brain disorders. ‘Brain disorders’ was the original term used by the PARADISE project for the group of neurological and psychiatric conditions it was addressing. When developing the rationale and research questions for the PARADISE project these conditions were selected because they are among the most prevalent of all brain disorders in Europe, they cross demographic and socioeconomic categories, and count as some of the most burdensome of all health conditions [7], [17], [18]. The diversity of these conditions provides the opportunity to explore commonalities across a wide range of conditions.

The operationalization of PSDs used in PARADISE and in this investigation is based on the conceptual model of the ICF as mentioned in the second paragraph of the introduction.

The qualitative data analyses for this investigation were performed in two stages: Firstly, meta-narratives were created for each of the conditions based on qualitative literature identified in systematic reviews and focus group data collected within the PARADISE project; secondly, a theory-driven thematic analysis of the narratives identified the PSDs, their facilitators and barriers. The aim of the narratives was to generate a rich account of the dynamic complexity of the lived experience of people with these health conditions. The thematic analysis of the narratives seeks to distil the PSDs, their facilitators and barriers and provide a vehicle for comparison between conditions.

### Systematic Literature Reviews

Literature reviews for all brain disorders relevant for this investigation were carried out in the PARADISE project [19]–[23]. Reviews for dementia and migraine were also conducted but excluded from this study because they had insufficient data to generate a narrative. For each review, two electronic searches in MEDLINE and PsychINFO databases, from 1 January 2005 to May 2010, were developed. Searches were limited to articles in English. Search terms were customized to each database and combined with MeSH headings for each brain disorder and with the following terms to identify studies addressing PSDs: “psychosocial*”, exp Quality of life/, exp Personal satisfaction/exp Human activities/exp social support/disabilit*, homelessness, environmental factor*, exp Interpersonal relations/, exp Quality of life/, exp personal satisfaction/, exp human activities/, exp paternalism/, prejudice/, psychosocial deprivation/, social values/, exp Social Problems/, Social Adjustment/, social isolation/stereotyping/, exp Social environment/, exp emotions/, exp family/, exp socioeconomic factors/exp life style/exp Disability evaluation/, exp Communication Barriers/, “Adaptation”, exp Psychological/, exp Aggression/, exp Psychological stress/, exp community (no microbial community)/, Sexual* or intimacy.

Studies were included if the study population fulfilled the diagnostic criteria agreed on for each brain disorder and were randomized control trials, control clinical trials, open intervention trials, longitudinal observational studies or qualitative studies and if they involved adult populations. The quality of the included studies was evaluated according to the National Institute for Health and Clinical Excellence [24]. The methodology followed in the systematic literature reviews is further reported by Cabello and colleagues [19].

### Focus Groups

For the focus groups carried out within the PARADISE project all study-related documents, as well as information on the consent procedure, were submitted to the Ethics Committees of the study centres prior to commencement of the study. All study centres received ethical approvals from their respective Ethics Committees (Ludwig-Maximilians-University, Munich, Germany; Hospital Universitario de la Princesa, Madrid, Spain; Fondazione IRCCS Istituto Neurologico “Carlo Besta”, Milan, Italy; Instytut Psychiatrii I Neurologii, Warsaw, Poland). The following inclusion criteria were applied to check participants’ suitability to enter the study: (1) an ICD-10 diagnosis of depressive disorder (F32.0, F32.1, F33.0, F33.1, F33.4), epilepsy (G40.0–G40.3), multiple sclerosis (G35), Parkinson’s disease (G20), schizophrenia (F20), or stroke (I60–I64), (2) sufficient cognitive capacity to participate in a focus-group session (score ≥26 in the Mini Mental Status Examination, (3) knowledge of the language in which the session was performed, as well as sufficient capacity to communicate, (4) age of at least 18 years; and (5) no additional severe mental disorder (‘comorbidity’). Those eligible were invited to participate and informed about the study, and their questions answered. Participants in the study gave written informed consent, pursuant to the Declaration of Helsinki 1996.

The sampling strategy was opportunistic, i.e., participants were the usual patients in the corresponding centres and were selected by their availability. Nonetheless, a maximum variation strategy regarding age and gender was used to ensure a wide range of views and perspectives. Focus group data of substance dependency was not used because the literature review was conducted only on alcohol dependency. Two researchers carried out the focus group sessions, one acting as moderator and the second as assistant. After introducing the objective of the study, the researchers performing the focus groups used a topic guide including open-ended questions to initiate the discussion. One example of these questions was ‘What are the difficulties and challenges you experience in everyday life?’ All sessions were digitally recorded using local languages. All researchers had training and/or previous experience in conducting focus groups. Qualitative data analyses were done in the respective language to identify and explore the significance of the PSDs experienced by the participants of the study as well as the factors associated with these PSDs. Further details about the focus group methodology is presented elsewhere [25]. The generated PSDs and associated factors resulting from the analyses were used as a basis to develop the qualitative narratives.

### Qualitative Narratives

The study employed a modification of the qualitative narrative analysis originally described by Todres [26], [27] and recently used by Wertz and colleagues [28]. This ‘composite first person narrative’ (CFPN) provides a reflective story about individual’s experiences by constructing a composite picture from participant’s self-reports. The method does not aim to be a mere re-telling of the evidence, but a narrative that reflects a richer and more evocative understanding of the complex experiences. It is grounded in: disciplinary experience, the literature about the phenomena under enquiry, listening and hearing the stories told by the participants and developing a narrative through reflection and interpretation. Todres notes that resulting narratives should connect with universal human qualities and enable the reader to relate personally to the events described: this is the strength of the approach. He argues that this satisfies the principles outlined by Malterud regarding relevance, validity and reflexivity of qualitative research data [29] and facilitates a deeper level of understanding of the topic. The narratives generated do not aim to be exhaustive and use personal pronoun ‘I’ as an essential part of the method because “… *it indicates the composite-informant in the first person sense as someone who typifies the general experience within a living and situated context*” (p. 5883) [28].

Wertz and colleagues only used primary participants’ self-reports when applying Todres method to develop narratives. This study uses in addition to self-reports from focus groups, evidence from qualitative literature identified through a systematic literature review. To distinguish the method used in this study from that of Todres and Wertz, the term ‘composite personal meta-narrative’ (CPMN) was adopted following the notion of ‘meta-narratives’ as described by Greenhalgh and colleagues [30] and on the basis that value is added through synthesising data from the literature together with new primary data from participants’ self-reports.

The process of generating the final version of each narrative by two researchers was as follows:

1. Two researchers were assigned to each brain disorder, one located in the country where focus groups were carried out and one in the UK. The two UK-based researchers worked independently across narratives and covered the seven conditions between them (SH- 4, MMcA- 3).
2. The ‘in-country’ researchers read the focus group transcripts in the local language.
3. Both researchers independently read the papers.
4. The pairs of researchers reflected on the data to gain familiarity with and knowledge of the key messages.
5. They independently generated a short, structured summary of the key messages addressing the research questions and a first draft of the narrative.
6. The pairs shared and agreed on key messages and these messages were subsequently divided according to PSDs, facilitators and barriers.
7. The pairs shared and agreed on the form and content of the narratives and edited them in an iterative process making sure that they reflected all agreed key messages until a final version was agreed upon.

Differences in interpretation in any step were addressed by re-examining articles for the phraseology and vocabulary and consulting colleagues.

### Theory-driven Thematic Analysis

The second stage of the analysis involved using the narratives themselves as a data source. Themes were developed using the method for interpretive synthesis called meta-ethnography [31] as described by Dixon-Woods and colleagues in the paper in which they introduce their review method as a “critical interpretative synthesis” [32]. This second stage provided an opportunity for deeper reflection and ‘back translation’ of the data, analogous to the standard process used in language translation to improve the reliability and validity of linguistic data [33]. The justification for using this method on data that has already been synthesised was that the process gave a further opportunity to increase an understanding of the significance of the narratives and an awareness of the key issues useful for a refinement of the interpretations. Arguably, the themes that remained constant through the forward and backward process are robust and provide a mechanism for comparison between the narratives. Two authors (SH and MMcA) independently listed key themes from the seven narratives. By means of discussion and negotiation a final agreed list of themes was produced which was then divided into two groups, PSDs and facilitators and barriers. The narrative data was jointly coded into this thematic structure.

## Results

### Systematic Literature Reviews

The seven systematic literature reviews identified a total of 1019 papers. From these, 77 (7.1%) papers used qualitative methodology with people with the mentioned brain disorders and were selected for this investigation. Information about the qualitative studies used to create the qualitative narratives is presented in Table S1.

### Focus Groups

In total, 45 persons participated in six focus group sessions. Information about the number of participants and where they were conducted is presented in Table S1.

### Qualitative Narratives

The narratives are presented here by health condition, first with an overview of the data used to generate the narrative, followed by the CPMN itself. Pre-edited versions of the narratives are available on request from the corresponding author. Further information about the aims and purposes of the qualitative studies used can be found in Appendix S1.

#### The experience of living with depression

From nine qualitative articles [4], [34]–[41] and one focus group two reviewers independently generated key messages and two drafts of a narrative on depression. They shared information and agreed on the key messages and on how to divide them into PSDs, determinants (barriers) and facilitators.

The generated determinants were: experience of adolescence, pregnancy, work, old age, male gender and society’s limited understanding of depression, including that of health professionals. The generated PSDs were: mood problems, social relationships, performance on daily activities, treatment related problems including re-enforcement of a sick identity. The generated facilitators were: trusting relationships that are a key to recovery, with families and self-help groups as the most important sources of support.

The following narrative was created in an iterative process from the first narrative drafts making sure that the agreed key messages were reflected.

#### Depression CPMN

When I was teenager I just knew that something was wrong with me because I cried or flipped out for no reason at all. I felt worthless and sad. I left my studies because I wasn’t able to concentrate and was left behind. I felt too ashamed to talk about emotional issues. I did go to see a doctor for help but he didn’t have time to listen and certainly didn’t understand, so I did not visit him anymore.

When I was first pregnant my partner was supportive, but later one we argued every day. I worried a lot about how I was going to cope financially, I didn’t sleep much and this made it all worse. I did have lots of other support, but couldn’t commit to it. It was weird. I didn’t feel proud or pleased and tried to hide my ‘bump’. I was sort of ashamed. Every time I went to see the midwife I got someone different. I didn’t want to tell my story over again, so after a while, I just didn’t tell it. When my partner eventually left me, my parents were wonderfully supportive, but I felt I was letting them down.

Things didn’t get better after my son was born. I realized I wasn’t going to be able to have the life I had planned. You try to look like you have it all together but inside you are like a pack of cards. I did not want my problems to affect the baby, but of course they did. Later when he went to school I got a job things were better, but as the years went by I started to get sick and took more time off work. My colleagues thought I was exaggerating, or even crazy. I felt so alone, I got bad headaches and backaches and was convinced I was seriously ill, but my doctor couldn’t find anything wrong. Another doctor thought I was depressed and offered me medication just like that! I was scared that I might become addicted to the pills so I just took them when I was desperate. I felt trapped, lost and out of control. I tried to throw myself into work, because when I was working, or with strangers, I felt a bit better.

When I was made redundant I was so scared, but at the same time relieved not to have the stress of work anymore. I spent all my time in bed. I started to think about ending my life, I lost all hope. Fortunately my family came to the rescue and arranged for me to see a counsellor. I began to realise that there might be help out there and I was angry with myself for leaving it so long. My counsellor ‘walked beside me’ for several years and I learned that when I was feeling low, the only way out was to talk. I joined a self-help group and was surprised at how comforting it was to know that other people felt the same way about things; the guys in the group seemed to have an even harder time sharing their problems. They felt irritable and angry more than sad.

The years passed; my son grew up, got married and moved away. I don’t see him often but he phones me to see if I am OK. Like many people my age, I spend a lot of time alone. All my friends have died now or moved away. I try not to bother the doctor too much. I struggle to get out of bed some mornings, simple tasks take me a long time and it’s difficult to make decisions, but I don’t tell the doctor about that, I don’t think he can understand what it is like to be old and live alone.

#### The experience of living with epilepsy

From three studies [42]–[44] and one focus group two reviewers independently generated key messages and two drafts of a narrative on living with epilepsy. They shared information and agreed on the key messages and on how to divide them into PSDs, determinants (barriers) and facilitators.

The generated determinants were often linked to limited knowledge about epilepsy. Lack of empathy by health care staff and the availability and cost of drugs were other concerns. The dominant PSDs were: fear, loneliness and limited social and work opportunities. The generated facilitators included better knowledge and understanding, recognition of the need for independence and the value of self-help groups and other social activities that promoted participation.

The following narrative was created in an iterative process from the first narrative drafts making sure that the agreed key messages were reflected.

#### Epilepsy CPMN

I knew there was something wrong with me but I didn’t know what. I would be sitting there watching TV with my family, zoning in and out several times a day. I was frightened and scared, and didn’t want to tell my Mum and Dad, but eventually they noticed themselves and took me to see the doctor. The diagnosis was a bit of a relief, at least my family stopped thinking I was making it up, but I felt lonely and was so unsure about lots of things. There was no step by step guidance. You can find information on the web, but sometimes you just want to forget it all.

I could tell my Mum and Dad were worried and that made it worse. Someone suggested that I attended a group, I didn’t want to go at first but really this was the best thing that happened to me at that time. It was so re-assuring to meet and talk to other people with similar problems but unfortunately my parents were embarrassed and didn’t understand. I know they worried a lot about the cost of the drugs. I didn’t really understand this until I grew up a bit and had to find the money myself. Getting the drugs wasn’t so bad for me, but I knew others who had a lot of difficulty.

I used to get really upset with the doctors who only had a few moments to see me, I was just longing to talk to someone who knew what it was like to live with this uncertainty and what I could do for the best. I felt so lonely! I was so upset and insulted when they classified me as ‘mentally ill’ so that I could be eligible for assistance. Did anyone ever think how this made me feel?! When the doctor suggested seizure surgery I was so scared.

After work I often sit by myself, the phone does not ring and I don’t go out often. I am not allowed to drive so can’t socialize much. People are so uneducated they think ‘oh my God, what happens if I come out with you and you have an attack?’. Having epilepsy envelopes you, you can’t seem to have a life, but others seem FREE. When I was 18 I thought it was time for me to be on my own, I refused to stay at home and went to college, but I couldn’t cope so I had to return. I felt so bad and no one understood. I hated feeling I was a burden to my parents so I went to work and now I support myself and live in the community. Getting a job was difficult. Once you have a seizure at work they find an excuse to ‘let you go’, but you HAVE to tell employers. I know everybody has something they have to struggle with but the public need more information about how it feels to live with epilepsy so they can understand and include people like me.

#### The experience of living with alcohol dependency

From twelve qualitative articles [45]–[56] two reviewers independently created two drafts of a narrative on alcohol dependency. They shared information and agreed on the key messages and on how to divide them into PSDs, determinants (barriers) and facilitators.

The generated determinants included poverty, homelessness, poor relationships, abuse (including sexual), social pressures and expectations, ignorance, available help and gender differences. The PSDs were a loss of control and self-respect, depression and despair and creating or perpetuating problems such as poverty, failed relationships, homelessness, unemployment, risky behaviours and HIV. Generated facilitators were friends and family support, treatment, education of others in the community, self-help groups, self-readiness and realization, recognition of the stages of indulgence, ambivalence and preparedness to construct alternative lives and responding appropriately at the appropriate time.

The following narrative was created in an iterative process from the first narrative drafts making sure that the agreed key messages were reflected.

#### Alcohol dependency CPMN

I began to drink alcohol when I was very young. I enjoyed drinking, it gave me an excuse to behave in an uninhibited way, but when I left school I had difficulty finding work and keeping it. I began to notice that I had lost control over my life. Once when I was homeless I ended up in a recovery community. The people were tolerant and supportive, but I felt controlled and I left because I thought that I could manage on my own. This was a mistake and I was soon back in my old ways. I felt I was not a ‘good person’, and that I didn’t deserve much from my life, my health was damaged.

My new partner asked me to take part in some AA meetings, but I was not ready for it, so soon stopped going. However, she did well and this made me hope for real change. Finally, I got help from a spiritual program. I found great help from others who had the same feelings, problems and challenges in life. I had reached a turning point – I really wanted the change. I have been abstinent for three years now. It has not been easy I still dream about drinking and I am afraid of a relapse. I have tried to find new friends and to rebuild relationships with my family. I have enjoyed being back at school and hope that one day I will get a good job because I am so tired of being poor.

Many of my former friends either did not join the spiritual program or dropped out, I learned not to keep in contact with them to avoid being ridiculed. I have one friend who quit drinking without getting any treatment. However, I don’t think he lost control in the same way as I did he always managed to keep his job.

My ex-wife is a sad story. Her parents had not taken good care of her, so she had not learned about healthy ways of living. She began drinking to overcome panic attacks and depression, We were under 20 when we met and I think we were both unprepared for independent life and felt overwhelmed by all the challenges. Our main entertainment was to drink. Our life was not good, we did not have work, we did not have money so we lost our apartment. She could not enjoy sex, because she had been abused as a child and by her former partner but we were happy when she got pregnant. For a while this really motivated her to get better for the sake of the baby. She tried to begin to build an alternative life, but she could not give up drinking and we had to give our child away. We divorced and she tried to commit suicide. She has tried to get treatment, but she does not have the inner power to quit. Nowadays, she prostitutes for her addiction and puts herself in very risky situations. I told her to attend a HIV/STI program to get some information –but she is the only one who can change her life.

#### The experience of living with multiple sclerosis

The PARADISE literature review identified 22 qualitative studies about multiple sclerosis (MS) [11], [57]–[77]. Three of these studies [58], [62], [63] were excluded because they focused on older people living with MS and on problems using wheelchairs and contained little information about PSDs. Two reviewers independently generated the narrative on MS from the remaining 19 references and one focus group. They shared information and agreed on the key messages and on how to divide them into PSDs, determinants (barriers) and facilitators.

The PSDs that negatively affected the achievement of desired roles in work and relationships often involved fear, frustration and uncertainty and reduced levels of participation. Also challenges to planning, pacing and prioritising together with communication issues such as not having needs understood and problems with articulation and flow of thought were identified as PSDs. Determinants were stigmatization, social exclusion, and invisible or actual environmental barriers including restricted mobility. Facilitators were identified as support from spouses and/or friends, formal support groups.

The following narrative was created in an iterative process from the first narrative drafts making sure that the agreed key messages were reflected.

#### MS CPMN

Before I was diagnosed I knew something was wrong but it took ages to find out what it was. I looked on the internet, watched TV and saw a poster in the GP surgery. I got to the stage where I just wanted to know. Admitting that I had MS was the biggest thing; I was devastated; I was 38 and I thought my life was over. I tried to keep it secret for as long as possible. I didn’t like the look on people’s faces when I told them. I think the hardest thing is not knowing what changes would happen next. I try not to think about how it was before, because I get too upset.

I really struggled to get the right help and information. The health professionals treat my symptoms, but the best ones are those that understand what I am going through and can provide quick, sensible simple suggestions. MS affects every part of my life, getting dressed, work, being a mother, a wife and friend. The tiredness is not like normal fatigue, it’s completely debilitating. I cried when I had to take early retirement as that was an important part of who I was. Not only did I have a lot less money, but I became isolated pretty quickly. It did give me more time to be with my young grandchildren but even that is a double-edged sword because they know you can’t do certain things, so they no longer ask.

The pain makes me feel depressed at times and stops me from thinking straight. I am worried that this together with my walking difficulties makes me look and sound drunk. I am frightened of falling. It is frustrating because you can’t be impulsive; everything has to be planned. I know I should pace myself because when I do too much, I really pay for it, but I can’t bear not being in control … being able to do what I want when I want. I feel people see me as a collection of symptoms or even just a wheel chair; the wheelchair definitely allows me to get out and about. Mind you, this also means that people talk over my head; I am restricted in where I can go and have to rely on someone to push me. A powered wheelchair would be good.

It is not all bad. It has brought my husband and me closer together, mind you the sex does nothing for me these days but he has needs and I want to meet them. There is always a balancing act though between losing independence and overburdening family and friends. I still go swimming with my friends but can only go to a pool that has a hoist and I don’t like being the centre of attention. You really get to know who your friends are. I don’t like it when my husband has to do the jobs that I think I should be doing; it makes me feel somehow diminished. The best thing is the friends I’ve met through the support group. They understand the frustration and loss of control, the constant battling with the disease, the incontinence, the shame, guilt, and the uncertainty. I belong to a different society, a disabled society. My body, my life doesn’t belong to me anymore; I have become ‘MS’ rather than someone with MS.

#### The experience of living with parkinson’s disease

From eleven qualitative articles [78]–[88] and one focus group two reviewers independently generated key messages and two drafts of a narrative on Parkinson’s disease. They shared information and agreed on the key messages and on how to divide them into PSDs, determinants (barriers) and facilitators.

The generated determinants were shock of diagnosis, difficulties concerned the physical aspects of Parkinson’s disease, including pain, reduced mobility, and reduction in ability for fine movements as needed for activities of daily living. These determinants, together with communication and swallowing difficulties, stress and anxiety, tended to affect socialization and productivity, and this often led to depression. It was noted that family members were affected by worry, embarrassment, or having to bear extra costs. The facilitating factors identified included therapeutic interventions that specifically targeted these difficulties as well as social and emotional support from friends and family.

The following narrative was created in an iterative process from the first narrative drafts making sure that the agreed key messages were reflected.

#### Parkinson’s disease CPMN

It began with some odd sensations; things didn’t smell or taste right, my GP referred me for an MRI scan: I went into the ‘iron pipe’ OK and came out with Parkinson’s disease! The diagnosis was a real bombshell and the neurologist just said he would meet up with me twice a year; I felt completely at sea. All I could think about was wheelchairs and an early death. In the back of my mind I thought I would wake up and it would all be a bad dream. At the start I wasn’t keen on taking any medication but when I forgot to take it I froze and that was a rude awakening. I got really depressed, but pills, support and sort of coming to terms with it has eased that somewhat. The unpredictability means I have lost confidence. Sometimes I can move quite freely but some things take me forever such as putting on socks or using my mobile. I can feel my wife itching to do things for me. I don’t blame her, it must be frustrating, but I would really like to do them for myself if I can. Mind you she has made some sensible suggestions like shaving later in the day so I don’t cut myself.

I kept my Parkinson’s a secret as long as I could and tried to keep working, but I felt guilty because I wasn’t pulling my weight so I gave up work. Then I felt lost, lonely, angry and useless. These feelings are not quite so bad now that I am keeping busy with support groups.

Communicating can be a challenge. My voice gets quieter and quieter as the conversation goes on and my tiny scribbly writing means that it is difficult for me to keep in touch with my daughter. She says I should email her but I can’t even hit the right key at times and I try not to ask for help as I want to stay independent and not be a burden.

Sometimes I take risks, like driving over to the allotment, digging a lot and then driving back. I know I shouldn’t, but if I don’t take care of the plants, there won’t be fresh veg to eat; and it helps with the finances. The change in income has changed our lifestyle and I worry that this is a strain for my wife, as she is the only wage earner now. I don’t know how we’ll manage financially if she has to give up work to look after me.

I am worried about falling; but and I can’t help drooling which is embarrassing and this plus the fear of choking, stops me from going out for a meal. I have to be careful where I go anyway and sometimes I reckon I see things… like shadows as if a cat was crossing and this makes me freeze. Just touching my leg can make it go again. My son is embarrassed by it but my wife says ‘so what’. Sex is OK but needs a bit of planning; I don’t know why my wife puts up with me really.

The information I have got over the years from the medical team was good but I would like to see them more often so I could get more information from them. I went to one PD support group but everybody seemed to be much older so I found a younger group and it is good to get together with people who understand what it is like. Someone said I should exercise but sometimes I can’t be bothered or feel too anxious. Sometimes I feel self-conscious at the gym. It is OK for my mates to say ‘Don’t worry about what other people think’ but I do shake a lot and look a bit blank when I am tired. Over time the numbers of pills have been increasing and I fear that the treatment is not as effective as it was. I dread what the future holds; I’ve just got to stay fit and healthy, exercise, take my medication and hope for a cure.

#### The experience of living with schizophrenia

From eleven qualitative articles [89]–[99] and one focus group two reviewers independently generated key messages and two drafts of a narrative on schizophrenia. They shared information and agreed on the key messages and on how to divide them into PSDs, determinants (barriers) and facilitators.

The generated determinants included the illness itself, lack of public knowledge and understanding, the media’s contribution to unhelpful negative public image, negative or ignorant attitudes of mental health service workers, lack of continuity and long term view and limited resources. The generated PSDs were stigma, marginalization, discrimination, social withdrawal, disengagement, loneliness, fear despair and helplessness, problems with relationships and interpersonal skills (affecting family, intimate and occupational relationships), frustrations with mental health services, problems with self-esteem and overprotection, un-met needs for social reciprocity, constancy, hope and understanding, problems with finding and keeping work and a place to live. The facilitating factors included providing empathetic physical and social spaces, such as living spaces, work spaces and routine environments, meaningful occupations, often outside the home such as exercise (noted to be both a way to socialize and become healthier), supported employment, trust, knowledge in advance of what is happening, training for health workers to listen more and work in partnership and family support.

The following narrative was created in an iterative process from the first narrative drafts making sure that the agreed key messages were reflected.

#### Schizophrenia CPMN

When I got schizophrenia my world changed just like that! It was like a life sentence of rejection and poverty. I became instantly different, omitted, excluded and vulnerable. The challenges include not only the illness itself, but also other people’s fear and ignorance. This is nurtured by the media who constantly blame mentally ill people for violence and killings. No wonder the public think that people with schizophrenia are dangerous and scurry away with fear on their faces when they meet you!

For a long time I didn’t know what was wrong with me, I felt so lonely and worthless. They ran lots of tests, and afterwards didn’t tell me anything, they didn’t explain what I had or what I no longer had. It felt like the Doctors didn’t really know how to help me, they just gave medicine for a ‘quick fix’. Also I ended up having to tell my story from the beginning each time as the doctors often changed and no one seemed to listen, one blamed Mum for my illness, which I thought was really cruel and unfair. Being in hospital is really traumatic, there is always the fear of involuntary commitment, you can’t talk freely, so you tell them what they want to hear and wait to be rescued. I think all health professionals should be taught listen more carefully and give clear information about what they think is the matter with you. That way we might learn to trust them and work out a better treatment together!

I used to live with my parents and that didn’t change when I got ill, but other things did. I didn’t see friends anymore, go to parties or have a date. They became indifferent; even my closest friend disappeared when I told him about my illness. Some people seem to think I am lazy and that I could stop being sick. I feel so alone, but also frustrated with myself because I also make a lot of effort to avoid people and events.

Even though my mum seemed ashamed of me when the illness started, she and my sister have always been there for me. Sometimes it seems they are the only ones who understand, although they do try to protect me too much, (like double checking if I took medicine). This makes me feel useless and depressed. I don’t think it is good for me either, because if I don’t learn to face my problems I will never know how to solve them.

When I am not well I need more rest and I just lay in bed, hours are empty, it’s really tedious. I can be completely blank and not know what to do. I suppose I know now that I will never work and achieve what I originally wanted to do in life, I lost my first job because people eventually found out I was ill. I feel helpless but I am trying to accept it. I would really like find a proper job. I heard from a friend that supported-employment programs can work.

Recently my life has improved a bit, I have moved away from home into independent accommodation. The housing is geared to people who have mental illness, so we have things in common. My case worker helps spur me on. He treats me as if I am another competent person and I like the daily routine. Some of the others who live there have some awful stories, especially the girls who seem to have had a really hard time, violence and all that. Sometimes we share stories and this is a good feeling. I think we help each other. Recently I decided to join an exercise class and I am getting a little bit fitter, just enough to get through the day. I feel like this programme has helped me get my second wind. I’ve lost some weight and am getting better at taking my medication.

#### The experience of living with stroke

From six qualitative studies [100]–[105] and one focus group two reviewers independently generated key messages and two drafts of a narrative on stroke. They shared information and agreed on the key messages and on how to divide them into PSDs, determinants (barriers) and facilitators.

The generated determinants included functional problems related to Activities of Daily Living, leisure and mobility restrictions, in particular walking and driving. Cognitive issues, over- solicitous families and the resulting reduction in motivation and independence were also generated from the data. The generated PSDs were lack of control, anger, guilt, stress on the family, low self-esteem, reduced socialising, poor sexual relationships, lack of understanding, uncertainty about the future, and work limitations. Facilitators were the ability to get out of the house to meet people and getting back to work.

The following narrative was created in an iterative process from the first narrative drafts making sure that the agreed key messages were reflected.

#### Stroke CPMN

I hadn’t been feeling too well the day I had the stroke and then ‘bang’ I was on the floor, unable to move and no-one to help me. When I stumble or fall now, it takes me back to that first time. I really tried to do everything I could to recover as quickly as possible and I was doing really well to start with. The treatment didn’t last long enough and I seem to have reached stalemate. I don’t seem to be getting any better now. It’s so frustrating and depressing as I know some people who had a stroke are back to normal now.

I used to be easy-going but now the slightest thing sets off my temper. Afterwards I sit back and wonder why did I do that? My partner does tend to do things for me, because it takes me a long time and she is frightened I will fall. If she could just give me a bit of space I wouldn’t feel so useless, and such a burden. She doesn’t sleep well at night because she keeps checking on me; I think she is worried that I’ll have another stroke. I am frightened of becoming dependent, with my family taking over and thinking for me. I have lost all my energy. My family say that the stroke has made me lazy but they don’t understand what it is like for me … it is an uphill daily struggle both physically and mentally. I do go to an exercise class which is good, a combination of getting out of the house, meeting people and the class itself. I feel calmer as well. Nine holes of golf using a golf buggy and being able to do more than sit and watch my friends bowling are my next goals.

I need to get back to work, so I can be the wage earner again and so I can get out from under my partner’s feet. I also have to pass my driving assessment. Driving the car is like an extension to my legs, without it I am standing still and stuck indoors. I feel guilty because I am not there as a partner. I enjoyed sex before and when you love someone you want to express yourself, but when you can’t it’s very difficult. I can’t think she still fancies me – what with my droopy face, my tottery walk, and the help she has to give me getting washed and dressed. At the moment she has also had to take up the DIY and garden jobs I used to do, but she doesn’t do them like I would, which I find frustrating.

As time goes on I have fewer and fewer visitors. Sitting at home is boring, I don’t concentrate as well as I did and struggle to read a book, write a letter or use the computer. Before the stroke I would have just jumped into the car and gone out for the night, but now I have to ask my partner to take me where I need to go. In any case I can’t cope with crowds I am frightened I might fall and break something and be in an even worse state that I am now. My partner goes out with her friends more for meals and the like. She must wonder what she is stuck with, but I have a much greater appreciation of her and my family now. I think I need to stop mourning the loss of the person I was.

### Theory-driven Thematic Analysis

The themes and grouping identified based on the thematic analyses fell into two major groups: those concerning PSDs and those illustrating the facilitators and barriers that were determinants of these difficulties.

Table S2 presents the twelve PSDs which were common across all conditions and which fell into four domains: *emotions and feelings, social relationships, work and financial status and self-perception*. A further domain emerged in all but one, namely *daily activities,* which refer to problems with activities of daily living, such as ‘*it can take me ages to do fiddly things, putting on socks, using my mobile phone…’ (Parkinson’s disease)*, with leisure activities *‘this drooling and the fear of choking stops me from going out for a meal- something we used to enjoy’ (Parkinson’s disease)* and communication ‘*I struggle with reading a book, writing a letter or using the computer’ (stroke)*. Additional categories under social relationships also emerged from the majority of the health conditions such as sex problems ‘*I enjoyed sex before … I can’t think she still fancies me*’ (stroke). Affirmation through continued support, *‘I don’t see him (son) often but he is very supportive and often phones me to see I am OK (depression)’.*

Table S3 presents the eight personal and environmental determinants that apply to all the health conditions. The results indicate that facilitators and barriers at opposite ends of a continuum of environmental impact: for example, access to self-help groups can be a facilitator while lack of access is considered a barrier. For this reason, the theory driven thematic analysis identified both facilitators and barriers as determinants of PSDs. In addition the eight common determinants, the data indicate a further six present in the majority of these health conditions. These are; retrieving and understanding information (or not): e.g. ‘*I really struggled to get the right help and information’* (MS); Diagnosis issues ‘*the diagnosis was a bit of a relief, at least may family stopped thinking I was making it up’ (epilepsy);* Family and friends having time and inclination to listen: ‘*what really made me suffer a lot was the indifference of my friends’ (schizophrenia);* Being valuable and making a contribution; ‘*Sometimes we share stories and this is a good feeling. I think we help each other.’ (schizophrenia);* Continuity of care: *‘I remember every time I went to see the midwife, I got someone different, and I just didn’t want to tell my story over again, so after a while I just didn’t.’ (depression); and Locus of control: ‘before my stroke I would have just jumped into the car but now I have to ask my wife to take me.’ (stroke).*

## Discussion

The most important contribution of this investigation refers to a new understanding of the psychosocial burden associated with brain disorders and how this can be addressed. We have provided evidence that there is a considerable degree of commonality in PSDs across the seven brain disorders studied. Since these were selected to represent brain disorders with different aetiology, symptomatology and trajectories, we would predict that the results also apply to other brain disorders.

Even though there is brain disorder-specific literature that shows that people with these disorders experience PSDs, such as the ones identified in this investigation (*Emotions and feelings*, *Social relationships, Work and financial status and Self-perception*) [106]–[109], no study has brought this together involving so many brain disorders so that the commonalities can emerge. The same applies to the determinants. There are studies for each brain disorder that emphasize the impact of different stages of life on the likelihood of experiencing PSDs [110]–[112], the importance of social networks and relationships for preventing deterioration [113] and the tremendous positive contribution that access to work can play for overcoming PSDs [114]. However, here we present first evidence that these determinants are relevant to a wide range of brain disorders.

The significance of this new understanding is that it opens the door to cross-cutting interventions requiring the involvement of different sectors. We have shown that the PSDs experienced by people with brain disorders are common and affect all areas of life. This implies that joint initiatives by a variety of agencies are required to address those PSDs regardless of the brain disorders. For example joint initiatives between the health sector and IT, financial, educational and social sectors, are needed for improving difficulties with communication, through the use of mobile phones, email and web browsing; for maintaining financial management skills, through book keeping training programmes and for improving self-confidence in the performance of activities of daily living, through counselling and social care. These can be organized centrally for people living with a variety of brain disorder with the advantage that resources and professional skills can be shared and potentially be implemented more effectively. This approach is, however, contrary to the general trend of providing highly specialized care and services framed around specific brain disorders often leading to duplication of services and reinforcing the condition specific silos.

The results of this study also challenge the idea that ‘nothing can be done’ when medical interventions are exhausted. We show that PSDs from the perspective of the person form their core experiences and therefore interventions targeting these difficulties can contribute tremendously to achieving a fulfilled life despite having a brain disorder. This concurs with the recommendations of the World Report on Disability [115], which also emphasises the importance of access to work and social, participatory approaches and illustrates the relevance of personal and environmental determinants in disability.

An additional contribution of this investigation relates to the methodology used to gain the understanding that PSDs are common across brain disorders. This methodology has the potential to be applied to other areas in which the person’s perspective is a central source of information. We introduce an innovative adaptation of a new method of analysis and illustrate how reports of previously completed studies can be used together with primary data from focus groups to produce narratives of lived experiences. It also illustrates how these narratives can be re-analysed to identify themes which draw out information often not highlighted in the original studies. For example, evidence around loss of control or fear of losing one’s autonomy is a major theme in this analysis but it is rarely mentioned in the original articles. This supports the view of Greenhalgh and colleagues [30], that extra value can be added by synthesising existing research.

The strength of the narrative part of the technique is that it provides the opportunity for the reader to empathise with people who have brain disorders. For any reader, but more importantly, for health workers and planners, relating these life experiences to their own, provides a vehicle for facilitating deeper levels of understanding between them and their clients, working towards a common understanding of the issues that really matter to those experiencing the health condition. This (or similar) effect of the narratives have been shown in Wertz and colleagues [28].

The two-stage analysis (narrative generation followed by thematic analysis of the narrative) also allows reflexivity and a deeper understanding of, in this instance, the complexity and change over time of PSDs. The re-analysis or ‘back analysis’ of the themes in the narrative further illustrates the complexity of the relationship between the determinants, PSDs, and environmental factors. For example the re-analysis data illustrates more clearly how access to work can have both a positive and a negative influence on people’s lives, on the one hand it can be overwhelming, contributing to a feeling of worthlessness and despair and on the other, it can be a life line back to an independent life. The power of using the thematic analysis of the generated narratives became apparent when it reviled the two dimensional nature of determinants as facilitators and barrier. It was only at this final step of the process that this result crystallised.

One important observation made during the study was that despite recognition in the literature that qualitative data is important for understanding patient’s views [116] and essential for developing sensitive policies and plans [13], the proportion of the literature reporting qualitative evidence was found to be lower than 10%.

This investigation has a number of limitations. Some of them relate to the focus groups data, which were conducted in medical settings by medically trained personal. We do not know if persons in other settings would have mentioned the same PSDs. Also, although the focus groups were recorded and transcribed, they were not entered into a computer assisted programme, which would have facilitated a higher level of accountability. They also were not translated due to limited resources to a common language and consequently were only analysed by one of the allocated researchers. We are nevertheless confident that the iterative process and discussion by pairs in all methodological steps, have contributed to mitigate the potential bias originated by those sources of bias.

Another limitation refers to the qualitative articles retrieved from the systematic literature review. We only considered studies that had been identified in the scope of the PARADISE project, which had predefined inclusion and exclusion criteria [19] and from that selected literature we selected only the qualitative studies. Even though this selection was based on the assumption that qualitative data would provide information about the actual experience of people living with these brain disorders, we cannot exclude the possibility that data from quantitative studies would have also been valuable. This may be the reason why certain aspects relevant to people’s lives, such as the role of leisure for people with alcohol abuse, or self-help groups for people after stroke, are absent from both the narratives and thematic analysis. In addition, we did not carry out any quality evaluation of the journal papers because we felt that this process might reduce too much the sources of information.

Finally, since our study aims guided the information we extracted from the data, only PSDs and their determinants were identified and the more positive elements of people’s lives were omitted. We are aware that we do not reflect all aspects of living with these health conditions into one narrative, and that we do not represent all possible views. The resulting narratives therefore, do not provide a comprehensive perspective of the burden of living with a brain disorder but only a partial glimpse into living with these conditions. The narratives should only be read considering that they do not relate to one person’s life but are a summation of many possible experiences.

## Conclusions

This investigation provides evidence by means of an innovative adaptation of an existing qualitative method that there is a considerable degree of commonality in PSDs and their environmental and personal determinants across brain disorders. This recognition opens the door to cross-cutting interventions requiring the involvement of different sectors.

## Supporting Information

Table S1 **Details of data sources.**
(DOC)

Table S2 **Psychosocial difficulties common across the seven health conditions.** AD Alcohol dependency, DE Depression, E Epilepsy, MS Multiple sclerosis, PD Parkinson’s disease, SCH Schizophrenia, ST Stroke.
(DOC)

Table S3 **Facilitators and barriers relating to the psychosocial difficulties associated with seven different health conditions.** AD Alcohol dependency, DE Depression, E Epilepsy, MS Multiple sclerosis, PD Parkinson’s disease, SCH Schizophrenia, ST Stroke.
(DOC)

Appendix S1 **Aims and purposes of the literature used to generate the narratives.**
(DOC)
