# Supplementary material for: Narratives Reflecting the Lived Experiences of People with Brain Disorders: Common Psychosocial Difficulties and Determinants
Source: PLoS One. 2014 May 7;9(5):e96890. doi: 10.1371/journal.pone.0096890 (PMC4013080; doi:10.1371/journal.pone.0096890)
Supplement: Table S1 — Details of data sources. (DOC) [file pone.0096890.s001.doc]

**Table S1. Details of data sources.**

| **Brain disorder** | **Literature reviews of qualitative studies** | | | | | | | | | | | | | **Focus group studies** | | |
| --- | --- | --- | --- | --- | --- | --- | --- | --- | --- | --- | --- | --- | --- | --- | --- | --- |
|  |  |  | **Methods data collection** | | | | **Participants of studies** | | | | | | |  | | |
|  | **Total articles (articles included (n)**  **Data bases [n]** | **Countries** | **Individual interviews (n)** | **Focus groups (n)** | **Participative observations (n)** | **24 hr Diary (n)** | **Total sample size (N)** | **Participants with brain disorder (n)** | **Carers/ family (n)** | **Health professionals ^a)^(n)** | **Age (range)** | **Gender(n)[f/] ^b)^** | **Focus groups (n)** | | **Participants (n)** | **Place** |
| **Depression** | 5+4 (9) [9] | New Zealand, Spain, UK, USA | 201 | 1 | - |  | 208 | 148 | 7 | 53 | 18-82 | 115/93 | 1 | | 7 | Spain |
| **Epilepsy** | 3 (3) [3] | Australia, Italy, UK, USA | 228 | 8 | - |  | 262 | 158 | 68 | 36 | 9-69 | 89/79 | 1 | | 6 | Italy |
| **Alcohol dependency** | 12 (12) [10] | Brazil, Canada, Sweden, Taiwan, UK, USA | 168 | 2 | 13 (1 study) |  | 185 | N/A | N/A | N/A | 17-69 | 87/90 | -- | | -- | -- |
| **Multiple sclerosis** | 22 (19) [19] | Australia, Norway, Sweden, UK, USA | 268 (15 studies) | 89 (4 studies) | -- |  | 357 | 318 | 32 | 7 | 20-75 | 241/106 | 1 | | 8 | Germany |
| **Parkinson’s disease** | 11 (11) [11] | Canada, France, Italy, Sweden, UK, USA | 238 |  | 171 |  | 452 | 409 | 37 | 8 | 39-88 |  | 1 | | 8 | Italy |
| **Schizophrenia** | 12 (11) [11] | Australia, Canada, Italy, Spain, USA | 74 (6 studies) | 13 (3 studies) | 2 | 20 (1 study) | 182 | 117 | 48 | 17 | 18-72 | 80/73 | 1 | | 8 | Poland |
| **Stroke/ CVA** | 8 (6) [6] | Canada, Germany, Sweden, UK, USA | 168 (6 studies) | -- | -- |  | 168 | 134 | 34 | - | 33-85 | 47/113 | 1 | | 8 | Germany |
| **Totals** | 71 [63] | 13 countries (7 EU) | 1179 | 112 | 205 | 20 | 1825 | 1284 | 226 | 121 | 9-88 | 659/554 | 6 | | 45 | 4 countries |

N/A Not available.

a) Including physicians and traditional practitioners.

b) Gender figures given when available, gender of carers and HP often omitted
