# Supplementary material for: Narratives Reflecting the Lived Experiences of People with Brain Disorders: Common Psychosocial Difficulties and Determinants
Source: PLoS One. 2014 May 7;9(5):e96890. doi: 10.1371/journal.pone.0096890 (PMC4013080; doi:10.1371/journal.pone.0096890)
Supplement: Table S2 — Psychosocial difficulties common across the seven health conditions. AD Alcohol dependency, DE Depression, E Epilepsy, MS Multiple sclerosis, PD Parkinson’s disease, SCH Schizophrenia, ST Stroke. (DOC) [file pone.0096890.s002.doc]

**Table S2. Psychosocial difficulties common across the seven health conditions.**

| **PSDs** | **Supporting quotations from the narratives** |
| --- | --- |
| **Emotions, feelings** | |
| Low mood, sadness, unhappiness | *‘The pain makes me feel depressed at times and stops me from thinking straight.’(MS) ‘I got really depressed but pills, support and sort of coming to terms with it has eased that somewhat.’ (PD)* |
| Loneliness, feeling lost | *‘For a long time I didn’t know what was wrong with me, I felt so lonely and worthless.’ (SCH) ‘I felt lost, lonely, angry and useless.’ (PD) ‘I felt lonely, I was so unsure about lots of things.’ (E)* |
| Anger and frustration | *‘I used to be easy going but now the slightest thing sets off my temper.’ (ST) ‘I began to realise that there might be help out there and I was angry with myself for leaving it so long.’ (DE)* |
| Fear | *‘I am frightened I might fall and break something and be in an even worse state that I am now.’ (ST) ‘I was frightened and scared, I didn’t want to tell my Mum and Dad.’ (E) ‘There is always the fear of involuntary commitment.’ (SCH)* |
| Guilt, shame and embarrassment | *‘...starting to feel guilty because I wasn’t pulling my weight so I gave up work.’ (PD) ‘I feel guilty because I am not there as a partner.’ (ST)* |
| Uncertainty about the future | *‘I realized I wasn’t going to be able to have the life I had planned.’ (DE) ‘Over time the numbers of pills have been increasing and I fear that the treatment is not as effective as it was.’ (PD)* |
| **Social relationships** | |
| Poor social relationships and skills | *‘As time goes on I have fewer and fewer visitors.’ (ST) ‘I am worried that the fuzzy- headed feeling and my difficulty with walking makes me look and sound drunk.’ (MS) ‘I make a lot of effort to avoid people and events.’ (SCH)* |
| **Work and financial status** | |
| Financial worries | *‘I need to get back to work, so I can be the wage earner again.’(ST) ‘Our life was not good, we did not have work, we did not have money, and we lost our apartment.’ (AD) ‘I am tired of being poor. (AD)* |
| Problems with work activities | *‘I lost my first job because people eventually found out I was ill.’ (SCH) ‘When I was made redundant I was so scared, but at the same time I was relieved not to have the stress of work anymore.’ (DE)* |
| **Self-perception** | |
| Worthlessness and loss of confidence | *‘The unpredictability means I have lost confidence.’ (PD) ‘I felt I was not a ‘good person’, and that I didn’t deserve much from my life.’ (AD)* |
| Loss of control | *‘I know I should pace myself because when I do too much, I really pay for it, but I can’t bear not being in control ... being able to do what I want when I want.’ (MS) ‘I began to notice that I had lost control over my life.’ (AD)* |
| Vulnerability | *‘With more time at home I lost all hope. I had to force myself to do anything. I spent all my time in bed. I started to think about ending my life.’ (DE)* |

AD Alcohol dependency, DE Depression, E Epilepsy, MS Multiple sclerosis, PD Parkinson’s disease, SCH Schizophrenia, ST Stroke.
