# Supplementary material for: Narratives Reflecting the Lived Experiences of People with Brain Disorders: Common Psychosocial Difficulties and Determinants
Source: PLoS One. 2014 May 7;9(5):e96890. doi: 10.1371/journal.pone.0096890 (PMC4013080; doi:10.1371/journal.pone.0096890)
Supplement: Table S3 — Facilitators and barriers relating to the psychosocial difficulties associated with seven different health conditions. AD Alcohol dependency, DE Depression, E Epilepsy, MS Multiple sclerosis, PD Parkinson’s disease, SCH Schizophrenia, ST Stroke. (DOC) [file pone.0096890.s003.doc]

**Table S3. Facilitators and barriers relating to the psychosocial difficulties associated with seven different health conditions.**

| **Facilitators and (*barriers)*** | **Supporting quotations from the narratives** |
| --- | --- |
| Stages of life and associated roles can act as both facilitators and barriers - adolescence, pregnancy, old age, gender and associated roles | *‘I left my studies because I wasn’t able to concentrate and was left behind as others moved forward...’ (DE) ‘We were happy when she got pregnant. For a while this really motivated her to get better for the sake of the baby…..’ (AD)* |
| Trusting or supportive relationships with individuals, family, health *professionals (lack of access to, and understanding by these groups)* | *‘I got help from a spiritual program. I found great help from others who had the same feelings, problems and challenges in life.’ (AD) ‘The neurologist just said he would meet up with me twice a year; I felt completely at sea.’ (PD) ‘He (Dr) didn’t have time to listen and certainly didn’t understand, so I did not visit him anymore.’ (DE)* |
| Social inclusion *(exclusion or rejection or stigma)* | *‘After work I often sit by myself, the phone does not ring and I don’t go out much. ... People are so uneducated they think ’oh my God, what happens if I come out with you and you have an attack?’ (E) ‘The challenges include not only the illness itself, but also other people’s fear and ignorance which is nurtured by the media who constantly look for sensation and blame mentally ill people for violence and killings. No wonder the public think that people with schizophrenia are dangerous and scurry away with fear on their faces when they meet you! ‘(SCH)* |
| Work opportunity *(limited or no work opportunity)* | *‘I tried to keep working, kept my Parkinson’s a secret as long as I could but I was starting to feel guilty because I wasn’t pulling my weight.’ (PD) ‘I need to get back to work, so I can be the wage earner again and so I can get out from under my partner’s feet. I don’t know if I’ll manage it.’ (ST)* |
| Self-help groups both formal and informal *(poor or lack of such groups)* | *‘The best thing is the friends I’ve met through the support group. They understand the frustration and loss of control, the constant battling with the disease, the incontinence, the shame, guilt, and the uncertainty.’ (MS) ‘Someone suggested that I attended a group, I didn’t want to go at first but really this was the best thing that happened to me at that time. It was so re-assuring to meet and talk to other people with similar problems.’ (E)* |
| Independence or self-determination/internal locus of control *(dependency external locus of control)* | *‘Recently my life has improved a bit, I have moved away from home into independent accommodation’. (SCH) ‘Before the stroke I would have just jumped into the car but now i have to ask my partner to take me where I need to go.’ (ST)* |
| Professionals having time to listen *(professionals without time or motivation)* | *‘My case worker helps spur me on. He treats me as if I am another competent person and I like the daily routine’. (SCH) ‘The best professionals are those that understand what I am going through and can provide quick, sensible simple solutions to my problems.’ (MS)* |
| Balance between protection and overprotection *(imbalance between them)* | *‘She (wife) doesn’t sleep well at night because she keeps checking on me; I think she is worried that I’ll have another stroke’. (ST) ‘I tried several ‘treatments’. Once when I was homeless I ended up in a recovery community. This was a good place, the people were tolerant and supportive, but I felt controlled and I left because I thought that I could manage to control my drinking on my own. This was a mistake and I was soon back in my old ways.’ (AD)* |

AD Alcohol dependency, DE Depression, E Epilepsy, MS Multiple sclerosis, PD Parkinson’s disease, SCH Schizophrenia, ST Stroke.
