# Supplementary material for: Narratives Reflecting the Lived Experiences of People with Brain Disorders: Common Psychosocial Difficulties and Determinants
Source: PLoS One. 2014 May 7;9(5):e96890. doi: 10.1371/journal.pone.0096890 (PMC4013080; doi:10.1371/journal.pone.0096890)
Supplement: Appendix S1 — Aims and purposes of the literature used to generate the narratives. (DOC) [file pone.0096890.s004.doc]

**Appendix S1. Aims and purposes of the literature used to generate the narratives.**

**Depression**

One article explored the relationship between patient’s views on their disorder and work and three articles analyzed management issues for depression in primary care settings. Another study sought to explain people’s perception of living with depression, and finally, four articles addressed the difficulties experienced by depressed people during critical periods in their lives.

**Epilepsy**

Two articles examined the experience of persons with epilepsy and their families, including knowledge about their condition, accessing epilepsy-related services and other health care, the lived experience of having epilepsy and the possible outcome trajectory after surgery; the third article analysed the psychosocial outcome after seizure surgery and the knowledge gaps and uncertainties about epilepsy as a disease affecting Chinese patients and their families.

**Alcohol dependency**

Four of the data bases were from Europe , five from USA , one each from Taiwan, Canada and Brazil . One of the USA studies was about native Indians . Four datasets had only women in which the relation between depression and alcohol were studied as well as in databases concerning both sexes . Two studies did not focus on the experiences of the addicted patients . The definition of an alcohol problem and the timing of the interview in relation to the phase of the alcohol problem varied. The two papers of Yeh and colleagues focused on abstinence in relation to moving from a lifestyle of using to one of not using.

**Multiple sclerosis**

The primary aims and purposes of the studies included investigating the experience of MS and related stigma , the meaning of fatigue and pain , perceptions of interventions and exercise , initial symptoms and diagnosis , effect on cognition and impact on daily life , care needs and services , changes in work and recreational activities , MS and women , sexual relationships and factors related to accidents .

**Parkinson’s disease**

One study conducted participant observations involving an unusually high number of patients (N=171) attending support groups and regional educational events . The primary aims and purposes of the studies were very varied and included: outlining the daily challenges for people with Parkinson’s disease and their families such as mobility, communication difficulties and psychological wellbeing such as stress, anxiety and depression. Facilitating factors refer to both interventions such as deep brain stimulation and psychological factors such as social support by specific groups of people, physical therapeutic interventions and palliative care.

**Schizophrenia**

The primary aims and purposes of these studies included exploration of stigma from the experience of people living with schizophrenia , the impact of therapeutic processes on their social lives and impact of exercise on health outcomes , investigating the process of transition to independence including supported employment , how social relationships are formed and maintained and how these affect daily occupation and participation in everyday life , and violence against women diagnosed with schizophrenia .

**Stroke**

The primary aims and purposes of the studies were to increase the understanding of the recovery process and effects of health care interventions and clarify what helps or hinders resumption of activities investigate the consequences of post stroke falls .

**References**

1. Millward LJ, Lutte A, Purvis RG (2005) Depression and the perpetuation of an incapacitated identity as an inhibitor of return to work. J Psychiatr Ment Health Nurs 12: 565-573.

2. Backenstrass M, Joest K, Rosemann T, Szecsenyi J (2007) The care of patients with subthreshold depression in primary care: is it all that bad? A qualitative study on the views of general practitioners and patients. BMC Health Serv Res 7: 10.1186/1472-6963-7-190.

3. Murray J, Banerjee S, Byng R, Tylee A, Bhugra D, et al. (2006) Primary care professionals' perceptions of depression in older people: a qualitative study. Soc Sci Med 63: 1363-1373.

4. Nolan P, Badger F (2005) Aspects of the relationship between doctors and depressed patients that enhance satisfaction with primary care. J Psychiatr Ment Health Nurs 12: 146-153.

5. Feely M, Long A (2009) Depression: a psychiatric nursing theory of connectivity. J Psychiatr Ment Health Nurs 16: 725-737.

6. Allan J, Dixon A (2009) Older women's experiences of depression: a hermeneutic phenomenological study. J Psychiatr Ment Health Nurs 16: 865-873.

7. Blanchard A, Hodgson J, Gunn W, Jesse E, White M (2009) Understanding social support and the couple's relationship among women with depressive symptoms in pregnancy. Ment Health Nurs 30: 764-776.

8. Kuwabara SA, Van Voorhees BW, Gollan JK, Alexander GC (2007) A qualitative exploration of depression in emerging adulthood: disorder, development, and social context. Gen Hosp Psychiat 29: 317-324.

9. Raymond JE (2009) 'Creating a safety net' women's experiences of antenatal depression and their identification of helpful community support and services during pregnancy. Midwifery 25: 39-49.

10. Sample PL, Ferguson PL, Wagner JL, Pickelsimer E, Selassie AW (2006) Experiences of persons with epilepsy and their families as they look for medical and community care: a focus group study from South Carolina. Epilepsy Behav 9: 649-662.

11. Wilson SJ, Bladin PF, Saling MM, Pattison PE (2005) Characterizing psychosocial outcome trajectories following seizure surgery. Epilepsy Behav 6: 570-580.

12. Snape D, Wang W, Wu J, Jacoby A, Baker GA (2009) Knowledge gaps and uncertainties about epilepsy: findings from an ethnographic study in China. Epilepsy Behav 14: 172-178.

13. Brown CG, Stewart SH (2008) Exploring perceptions of alcohol use as self-medication for depression among women receiving community-based treatment for alcohol problems. J Prev Interv Community 35: 33-47.

14. Dyson J (2007) Experiences of alcohol dependence: a qualitative study. J Fam Health Care 17: 211-214.

15. Jakobsson A, Hensing G, Spak F (2005) Developing a willingness to change: Treatment-seeking processes for people with alcohol problems. Alcohol and Alcoholism 40: 118-123.

16. Webb H, Rolfe A, Orford J, Painter C, Dalton S (2007) Self-directed change or specialist help? Understanding the pathways to changing drinking in heavy drinkers. Addict Res Theory 15 85-95.

17. Bezdek M, Spicer P (2006) Maintaining abstinence in a northern plains tribe. Med Anthropol Q 20: 160-181.

18. Brems C, Dewane S (2007) Hearing consumer voices: planning HIV/sexually transmitted infection prevention in alcohol detoxification. J Assoc Nurses AIDS Care 18: 12-24.

19. Sobczak JA (2009) Struggling to reconnect: Women's perspectives on alcohol dependence, violence, and sexual function. J Am Psychiatr Nurses Assoc 14: 421-428.

20. Sobczak JA, (2007) Managing depressive symptoms in the context of abstinence: findings from a qualitative study of women. Perspect Psychiatr Care 43: 84-92.

21. Wilton R, Deverteuil G (2006) Spaces of sobriety/sites of power: examining social model alcohol recovery programs as therapeutic landscapes. Soc Sci Med 63: 649-661.

22. Schlichting S, Boog MC, Campos CJ (2007) Lunchtime as a therapeutic moment: a health education approach with alcohol-dependent women. Rev Lat Am Enfermagem 15: 384-390.

23. Yeh MY, Che HL, Lee LW, Horng FF (2008) An empowerment process: successful recovery from alcohol dependence. J Clin Nurs 17: 921-929.

24. Yeh MY, Che HL, Wu SM (2009) An ongoing process: a qualitative study of how the alcohol-dependent free themselves of addiction through progressive abstinence. BMC Psychiatry. 2009/11/26 ed. pp. 76.

25. Borkoles E, Nicholls AR, Bell K, Butterly R, Polman RCJ (2008) The lived experiences of people diagnosed with multiple sclerosis in relation to exercise. Psychol Health 23: 427-441.

26. Finlayson M, Van Denend T, DalMonte J (2005) Older adults' perspectives on the positive and negative aspects of living with multiple sclerosis. Br J Occup Ther 68: 117-124.

27. Fong T, Finlayson M, Peacock N (2006) The social experience of aging with a chronic illness: perspectives of older adults with multiple sclerosis. Disabil Rehabil 28: 695-705.

28. Grytten N, Maseide P (2005) 'What is expressed is not always what is felt': coping with stigma and the embodiment of perceived illegitimacy of multiple sclerosis. Chronic Illn 1: 231-243.

29. Grytten N, Maseide P (2006) 'When I am together with them I feel more ill.' The stigma of multiple sclerosis experienced in social relationships. Chronic Illn 2: 195-208.

30. Malcomson KS, Lowe-Strong AS, Dunwoody L (2008) What can we learn from the personal insights of individuals living and coping with Multiple sclerosis? Disabil Rehabil 30: 662-674.

31. Douglas C, Windsor C, Wollin J (2008) Understanding chronic pain complicating disability: finding meaning through focus group methodology. J Neurosci Nurs 40: 158-168.

32. Olsson M, Lexell J, Soderberg S (2005) The meaning of fatigue for women with multiple sclerosis. J Adv Nurs 49: 7-15.

33. Boss TM, Finlayson M (2006) Responses to the acquisition and use of power mobility by individuals who have multiple sclerosis and their families. Am J Occup Ther 60: 348-358.

34. Dodd KJ, Taylor NF, Denisenko S, Prasad D (2006) A qualitative analysis of a progressive resistance exercise programme for people with multiple sclerosis. Disabil Rehabil 28: 1127-1134.

35. Smith C, Hale L, Olson K, Schneiders AG (2009) How does exercise influence fatigue in people with multiple sclerosis? Disabil Rehabil 31: 685-692.

36. Isaksson AK, Ahlstrom G (2006) From symptom to diagnosis: illness experiences of multiple sclerosis patients. J Neurosci Nurs 38: 229-237.

37. Shevil E, Finlayson M (2006) Perceptions of persons with multiple sclerosis on cognitive changes and their impact on daily life. Disabil Rehabil 28: 779-788.

38. Isaksson AK, Ahlstrom G (2008) Managing chronic sorrow: experiences of patients with multiple sclerosis. J Neurosci Nurs 40: 180-191.

39. Sweetland J, Riazi A, Cano SJ, Playford ED (2007) Vocational rehabilitation services for people with multiple sclerosis: what patients want from clinicians and employers. Mult Scler 13: 1183-1189.

40. Wollin JA, Yates PM, Kristjanson LJ (2006) Supportive and palliative care needs identified by multiple sclerosis patients and their families. Int J Palliat Nurs 12: 20-26.

41. Yorkston KM, Baylor CR, Klasner ER, Deitz J, Dudgeon BJ, et al. (2007) Satisfaction with communicative participation as defined by adults with multiple sclerosis: a qualitative study. J Commun Disord 40: 433-451.

42. McCabe MP, Roberts C, Firth L (2008) Work and recreational changes among people with neurological illness and their caregivers. Disabil Rehabil 30: 600-610.

43. Olsson M, Lexell J, Soderberg S (2008) The meaning of women's experiences of living with multiple sclerosis. Health Care Women Int 29: 416-430.

44. Prunty M, Sharpe L, Butow P, Fulcher G (2008) The motherhood choice: themes arising in the decision-making process for women with multiple sclerosis. Mult Scler 14: 701-704.

45. Esmail S, Munro B, Gibson N (2007) Couple's experience with multiple sclerosis in the context of their sexual relationship. Sex Disabil 25: 163-177.

46. Nilsagard Y, Denison E, Gunnarsson LG, Bostrom K (2009) Factors perceived as being related to accidental falls by persons with multiple sclerosis. Disabil Rehabil 31: 1301-1310.

47. Solimeo S (2008) Sex and gender in older adults' experience of Parkinson's disease. J Gerontol B Psychol Sci Soc Sci 63: S42-S48.

48. Buizza C, Schulze B, Bertocchi E, Rossi G, Ghilardi A, et al. (2007) The stigma of schizophrenia from patients' and relatives' view: A pilot study in an Italian rehabilitation residential care unit. Clin Pract Epidemiol Ment Health 3: 10.1186/1745-0179-3-23.

49. Gonzalez-Torres MA, Oraa R, Aristegui M, Fernandez-Rivas A, Guimon J (2007) Stigma and discrimination towards people with schizophrenia and their family members. A qualitative study with focus groups. Soc Psychiatry Psychiatr Epidemiol 42: 14-23.

50. Fogarty M, Happell B (2005) Exploring the benefits of an exercise program for people with achizophrenia: A qualitative study. Issues Ment Health Nurs 26: 341-351.

51. Moll MF, Saeki T (2009) Social life of people with diagnosis of schizophrenia, attended at a psychosocial care center. Rev Lat Am Enfermagem 17: 995-1000.

52. Hill A, Mayes R, McConnell D (2010) Transition to independent accommodation for adults with schizophrenia. Psychiatr Rehabil J 33: 228-231.

53. Liu KW, Hollis V, Warren S, Williamson DL (2007) Supported-employment program processes and outcomes: experiences of people with schizophrenia. Am J Occup Ther 61: 543-554.

54. Bejerholm U, Eklund M (2006) Engagement in occupations among men and women with schizophrenia. Occup Ther Int 13: 100-121.

55. Lencucha R, Kinsella EA, Sumsion T (2008) The formation and maintenance of social relationships among individuals living with schizophrenia Am J Psychiatr Rehabil 11: 330-335.

56. Yilmaz M, Josephsson S, Danermark B, Ivarsson AB (2008) Participation by doing: Social interaction in everyday activities among persons with schizophrenia. Scand J Occup Ther 15: 162-172.

57. Yilmaz M, Josephsson S, Danermark B, Ivarsson AB (2009) Social processes of participation in everyday life among persons with schizophrenia. Int J Qual Stud Health Well-Being 4: 267-279.

58. Rice E (2008) The invisibility of violence against women diagnosed with schizophrenia: a synthesis of perspectives. Adv Nurs Sci 31: E9-E21.

59. Carin-Levy G, Kendall M, Young A, Mead G (2009) The psychosocial effects of exercise and relaxation classes for persons surviving a stroke. Can J Occup Ther 76: 73-80.

60. Green TL, King KM (2009) Experiences of male patients and wife-caregivers in the first year post-discharge following minor stroke: a descriptive qualitative study. Int J Nurs Stud 46: 1194-1200.

61. Robison J, Wiles R, Ellis-Hill C, McPherson K, Hyndman D, et al. (2009) Resuming previously valued activities post-stroke: who or what helps? Disabil Rehabil 31: 1555-1566.

62. Schmid AA, Rittman M (2009) Consequences of poststroke falls: Activity limitation, increased dependence, and the development of fear of falling. Am J Occup Ther 63: 310-316.
